# Position-Specific Carbon Isotope Constraints on Propane Formation from Kerogen during Open-System Pyrolysis

**DOI:** 10.1021/acsomega.6c08660

**Published:** 2026-10-12

**Authors:** Yuanyuan Ma, Li Lu, Cheng Tao, Jipeng Li, Luyun Li, Huamin Yang, Yongge Sun

**Affiliations:** † Wuxi Research Institute of Petroleum Geology, Petroleum Exploration and Production Research Institute, 12377 SINOPEC, Wuxi, Jiangsu 214126, China; ‡ State Key Laboratory of Shale Oil and Gas Enrichment Mechanisms and Effective Development, SINOPEC, Beijing, 100083, China; § Key Laboratory of Petroleum Accumulation Mechanisms, SINOPEC, Wuxi 214126, China; ∥ Organic Geochemistry Unit, School of Earth Sciences, Zhejiang University, Hangzhou, Zhejiang 310058, China

## Abstract

Position-specific carbon isotopic compositions of propane have been proven to be a powerful tool for identifying the processes involved in natural gas generation. However, current understanding of intramolecular isotope distributions in propane and their geochemical implications is mainly derived from kerogen pyrolysis in closed-system experiments. In this study, open-system pyrolysis experiments were performed to investigate changes in intramolecular carbon isotope distributions of propane with increasing thermal maturation. Given the low abundance of propane in pyrolysis products, an offline enrichment protocol combining alkaline CO_2_ scrubbing and cryogenic trapping was developed. Coupled with GC-pyrolysis-GC-IRMS, this experimental setup was applied to collect and measure position-specific carbon isotopes of propane from a gas sample with a dryness coefficient up to 0.99. Open-system pyrolysis of a type II kerogen reveals that the isotopic difference between the central and terminal carbon atoms of propane increases from 2.7‰ to 6.7‰ across the oil window (Easy%*R*_0_ = 0.6–1.6%), followed by a decrease to 2.8‰ at the overmature stage (Easy%*R*_0_ = 1.8–2.6%). In contrast to the monotonic increase observed during closed-system pyrolysis, open-system pyrolysis yields a unique intramolecular isotope trajectory. This pattern is further supported by the correlation between δ^13^C_propane_ and δ^13^C_central_. With respect to the secondary cracking of propane at high- to overmature stages, pure-propane cracking experiments constrain the onset of substantial secondary cracking at approximately 520 °C (Easy%*R*_0_ = 1.9%), which provides a thermal reference for discriminating primary generation signals from secondary overprinting. The findings of this study provide a valuable complement to previous closed-system pyrolysis-based investigations and establish a framework for interpreting intramolecular isotope signatures of propane generated during kerogen thermal degradation in petroliferous basins.

## Graphical abstract

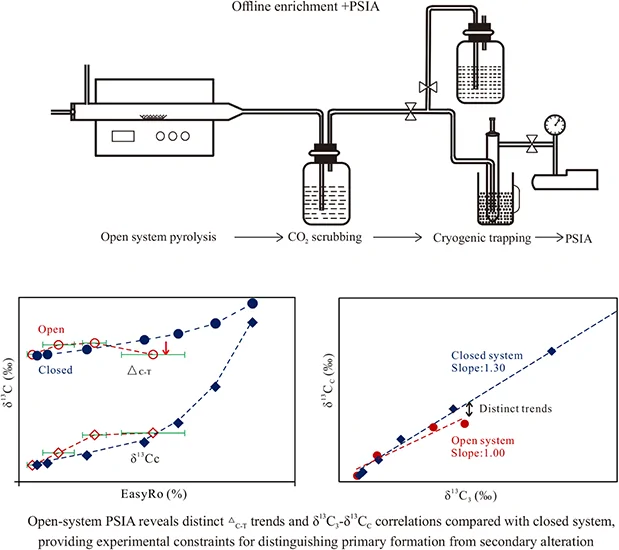

## 1 Introduction

Natural gas is a cornerstone of the global energy supply, and understanding its formation mechanisms is essential for reliable resource assessment and effective exploration. Conventional geochemical approaches—including molecular composition, bulk carbon and hydrogen stable isotope ratios—offer valuable insights, but interpretations may become ambiguous when gases originate from mixed sources or have undergone complex thermal histories.^1,2^ This is because such bulk approaches provide only overall compositions and average isotopic signals across all carbon positions. New analytical methods are therefore required. Position-specific carbon isotope analysis (PSIA) of propane has emerged as a promising approach. As the smallest hydrocarbon molecule with chemically nonequivalent carbon positions, propane provides intramolecular isotopic information that offers refined constraints on gas generation pathways, source characteristics, and thermal evolution.^1–6^

Several analytical techniques have been developed for PSIA of propane, including enzymatic conversion coupled with compound-specific isotope analysis, online GC–pyrolysis–GC–IRMS, high-resolution methods, quantitative ^13^C NMR, and mid-IR laser absorption spectroscopy.^7–11^ Among these, GC–pyrolysis–GC–IRMS has been extensively applied because it requires relatively small sample volumes and minimal preparation. Accordingly, PSIA has been increasingly applied to both laboratory pyrolysis products and natural gases.^12^ Propane can exhibit intramolecular carbon isotopic rollover, shifting from δ^13^C_central_ < δ^13^C_terminal_ to δ^13^C_central_ > δ^13^C_terminal_ with increasing maturity.^3^ This rollover reflects a shift in propane generation pathways,^4^ and kinetic isotope effects indicate that position-specific isotope distributions can distinguish such pathways.^3,4^ To further constrain propane generation signatures, laboratory pyrolysis experiments have been widely conducted.

Closed-system pyrolysis experiments (e.g., gold-tube, hydrous, and anhydrous pyrolysis) using kerogen, crude oil, and *n*-alkanes have improved the understanding of propane isotope systematics during thermal maturation.^1,5,13–15^ In such experiments, generated products are retained within the reaction system, allowing secondary cracking of early formed hydrocarbons to progressively modify primary generation signals as thermal stress increases.^16–18^ Consequently, PSIA signatures of propane from closed systems may represent mixed isotopic characteristics, potentially obscuring those associated with primary kerogen cracking.^5^ By contrast, open-system pyrolysis continuously removes generated products, thereby minimizing secondary reactions and preserving isotopic signatures more representative of primary formation processes.^18–21^ Despite the widespread application of open-system techniques (e.g., Rock-Eval pyrolysis and thermogravimetric analysis) to bulk and macromolecular products,^22–26^ their use for PSIA of propane remains limited. The primary challenge lies in collecting sufficient propane of analytical purity from transient pyrolysis products highly diluted by carrier gases and accompanied by CO_2_ and unsaturated hydrocarbons.^20,24,25^

To address low-concentration propane detection, Liu et al.^27^ developed an online hydrocarbon gas concentration system for natural gas samples, enabling PSIA of propane at concentrations as low as 0.005%. However, designed for relatively simple natural gas matrices, this system is less suitable for open-system pyrolysis products, in which the chemically complex matrix can interfere with chromatographic separation and compromise the purity of the isolated propane required for PSIA.

In this study, open-system pyrolysis was performed on a type II kerogen to investigate the evolution of propane generated during thermal degradation across varying thermal maturities. PSIA of propane, enabled by an offline enrichment method for trace-level propane, was undertaken to examine how intramolecular carbon isotope distributions vary with maturity, and the results were compared with those derived from closed-system experiments. Complementary pure-propane cracking experiments supplied a thermal constraint on secondary cracking. Collectively, these findings provide new position-specific carbon isotope constraints on propane formation from kerogen during open-system pyrolysis, advancing our understanding of hydrocarbon generation.

## 2 Materials and Methods

### 2.1 Materials

A certified propane standard gas (99.9% purity; Wuhan Newradar Special Gas Co., Ltd., China) was used both for method validation and for pure-propane cracking experiments to constrain the thermal onset of secondary reactions. A certified multicomponent reference gas (HB; Dalian Special Gases Co., Ltd., China) containing CH_4_ (2.04%), C_2_H_6_ (1.05%), C_2_H_4_ (1.10%), C_3_H_8_ (1.03%), C_3_H_6_ (1.06%), and H_2_ (1.07%) in helium balance was employed to evaluate the enrichment protocol for a suite of hydrocarbon components. All gases were supplied in passivated stainless-steel cylinders to minimize surface adsorption.

The GBW 04480 carbon and hydrogen isotope reference material of natural gas (China Petroleum Exploration and Development Research Institute, Langfang Branch) was utilized for routine instrument calibration and analytical accuracy monitoring.

A natural dry-gas sample (ZY1HF) from the Sichuan Basin, China, was used to test the enrichment protocol for highly mature natural gas with trace-level propane. In addition, kerogen isolated from the Late Triassic Chang 7 black mudstone (Sample YC)^28^ of the Ordos Basin, China, following the procedure of Zhang et al.,^29^ was used for both open- and closed-system pyrolysis experiments. Materials used in this study are summarized in Table 1.

**Table 1:** Sample Information

Sample	Description	Type
C3	Pure propane	Gas
HB	Standard gas mixture	Gas
GBW04480	Isotope reference material of natural gas	Reference gas
YC	Black mudstone, Ordos Basin (Upper Triassic)	Rock
ZY1HF	Natural gas, Sichuan Basin (Wufeng-Longmaxi Fm.)	Natural gas

### 2.2 Open-System Pyrolysis, Propane Enrichment, and Closed-System Pyrolysis

Open-system pyrolysis of the isolated kerogen (Sample YC) was performed using a custom-built apparatus comprising a tubular furnace, a gas-scrubbing unit, a cryogenic trap, and a vacuum line (Figure 1). Approximately 300 mg of kerogen powder was placed in a quartz boat at the center of a quartz tube reactor (20 mm i.d. × 600 mm) (OTF-1200X-S, Hefei Kejing, China; temperature precision ±1 °C). Samples were ramped from room temperature to 564 °C at a heating rate of 2 °C min^–1^ under a continuous flow of high-purity helium (99.999%) at 60 mL min^–1^. Pyrolysis gases were collected over four temperature intervals (370–404 °C, 416–466 °C, 476–500 °C, and 514–564 °C), corresponding to progressive maturity stages. Continuous gas removal during heating simulates natural hydrocarbon expulsion, in contrast to closed-system experiments, in which accumulated products undergo secondary reactions. For these open-system gas fractions, maturity was assessed for each temperature interval by applying the programmed time–temperature history to the Easy%*R*_0_ calculation.^30^ Since each gas fraction was collected over a finite temperature range rather than at a single isothermal temperature, the calculated Easy%*R*_0_ is expressed as a range defined by the onset and termination temperatures of each interval (Table S1). For plotting, the midpoint Easy%*R*_0_ of each interval was used, with the full range shown as horizontal error bars in the relevant figures.

**Figure 1:**
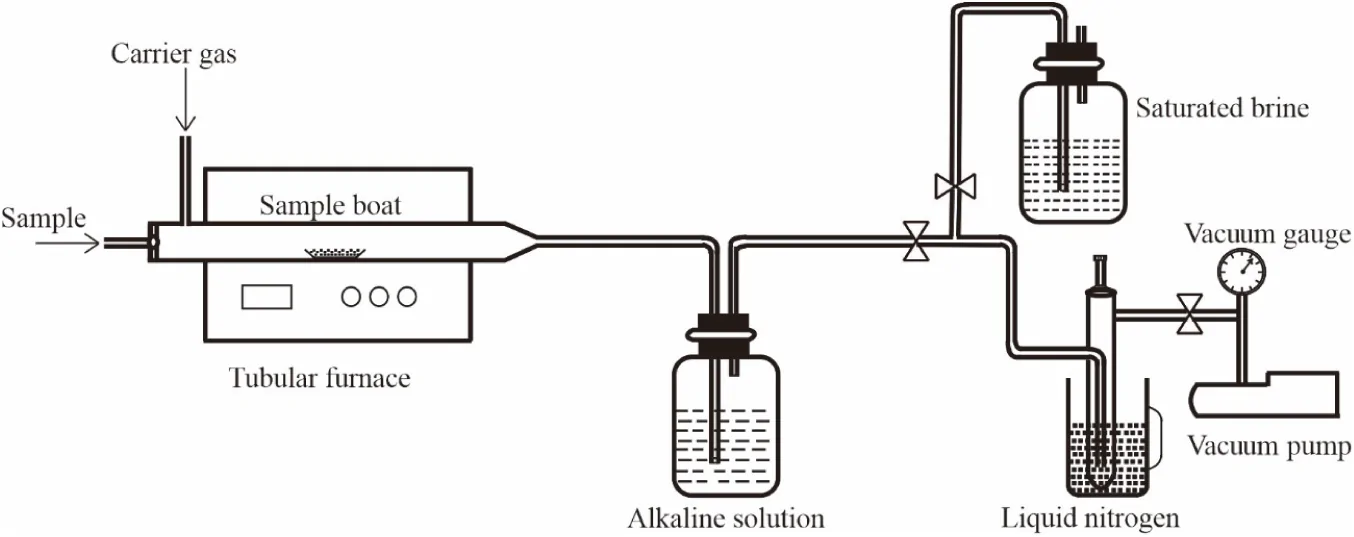
Schematic of the open-system pyrolysis setup.

Propane was enriched from the bulk pyrolysis gases using an offline cryogenic trapping protocol. The effluent gas stream first passed through a scrubbing bottle containing a 30% (w/v) KOH aqueous solution to remove CO_2_. The CO_2_-free gas was then transferred through polytetrafluoroethylene (PTFE) tubing (6 mm o.d., 4 mm i.d.) to minimize hydrocarbon adsorption, and subsequently condensed in a tube submerged in liquid nitrogen for 10 min. Noncondensable gases were removed by evacuating the trap to 60 Pa. The cold trap was then removed, and saturated brine was injected to flush the enriched sample into the neck of a collection vial for subsequent isotope analysis. The same protocol was applied to natural gas samples (ZY1HF) to validate its effectiveness for dry-gas matrices.

For comparison, closed-system pyrolysis experiments were conducted using gold-tube reactors, following the protocols of Zhao et al.^31^ with modifications for intramolecular isotope analysis. The same Type II kerogen isolate (sample YC, Table 1) used in the open-system experiments was employed. Briefly, kerogen aliquots (45–65 mg) were loaded into gold tubes (40 mm length, 4 mm I.D.). After purging with argon for 10 min, the tubes were sealed by arc welding under an argon atmosphere. The sealed tubes were placed in stainless-steel autoclaves and heated in an electric oven. Seven separate pyrolysis runs were carried out, each targeting a different final temperature: 336, 360, 408, 456, 480, 504, and 528 °C. For each run, the temperature was heated from room temperature to the target at 2 °C h^–1^, with a constant confining pressure of approximately 50 MPa maintained by pumping water into the autoclaves. Upon reaching the target temperature, the autoclave was immediately removed and quenched in cold water to terminate the reaction. For each closed-system run, Easy%*R*_0_ was calculated at each target temperature using the same kinetic model of Sweeney and Burnham.^30^

### 2.3 Validation of the Enrichment Protocol

The repeatability and accuracy of the enrichment protocol were validated by comparative δ^13^C analysis of enriched and nonenriched samples, which included both pure propane and a certified multicomponent reference gas (HB).

For propane validation, direct injection (5 μL) was compared with samples processed through the LN_2_/KOH trapping system (1 mL injection volume, 5 μL final injection, n = 3), and the recovery of this trapping system was 88% (see Table S2). For the HB reference gas, direct analysis (50 μL injection) was contrasted with enriched samples obtained by processing 50 and 200 mL volumes; both procedures yielded a postenrichment injection volume of 5 μL.

### 2.4 Propane Cracking Experiments

To independently constrain the thermal stability of propane and the onset of secondary cracking, we performed a sequential isothermal cracking experiment. The apparatus was heated from room temperature to 300 °C at a ramp rate of 2 °C min^–1^ with a continuous helium flow of 60 mL min^–1^ (identical to Section 2.2). Once the temperature stabilized, pure propane standard gas was introduced into the system. The generated C_2+_ hydrocarbon gases were collected by cryogenic trapping. The temperature was then successively raised to 500, 520, 550, and 600 °C at the same heating rate, and the injection-collection cycle was repeated at each temperature. All collected gas samples were analyzed for compound-specific carbon isotope ratios. These experiments provide a thermal reference to interpret the intramolecular isotope redistribution observed during kerogen pyrolysis.

### 2.5 Isotopic Analysis

Compound-specific carbon isotope analysis (CSIA) was carried out on an Agilent 7890A GC coupled to a Thermo Fisher Delta V Advantage IRMS. Hydrocarbons were resolved on an HP-PLOT Q column (30 m × 0.32 mm × 20 μm) with helium as the carrier gas. The column temperature was held at 40 °C for 1 min, then raised at 10 °C min^–1^ to 150 °C (held 1 min), and finally raised at 20 °C min^–1^ to 250 °C (held 5 min). The injector temperature was set to 110 °C. The separated compounds were combusted at 960 °C to CO_2_ before entering the IRMS for isotopic determination.

PSIA of propane was performed on a GC–Py–GC–IRMS platform as described previously.^32^ Propane was directed through a four-port valve into a pyrolysis furnace maintained at 820 °C, where it was pyrolyzed. The resulting pyrolysis fragments, primarily CH_4_, C_2_H_4_, C_2_H_6_, and C_3_H_6_, were resolved on a secondary GC (Trace GC Ultra). They were monitored simultaneously by flame ionization detector (FID) and IRMS for molecular composition and carbon isotope ratios, respectively. δ^13^C_terminal_ and δ^13^C_central_, along with Δ_C–T_ (δ^13^C_central_ – δ^13^C_terminal_), were derived from fragment isotopic data using the mass-balance equations of Li et al.^33^ Instrument performance was checked against GBW 04480 reference gas.

All δ^13^C data are reported relative to the Vienna Pee Dee Belemnite (VPDB) standard. Duplicate analyses yielded a precision better than ±0.4‰.

## 3 Results and Discussion

### 3.1 Validation of the Enrichment and Analytical Workflow

The reliability of the offline enrichment protocol was assessed using standard gases. Carbon isotope values of bulk propane (δ^13^C_propane_) and position-specific parameters (δ^13^C_terminal_, δ^13^C_central_, and Δ_C–T_) were obtained by direct injection and LN_2_/KOH trapping-based preconcentration; all offsets were within ±0.5‰ (Tables 2, 3; detailed replicate data in Table S3). After trapping, the precision for propane was ±0.3‰ (δ^13^C_propane_), ±0.2‰ (δ^13^C_terminal_), ±0.4‰ (δ^13^C_central_), and ±0.6‰ (Δ_C–T_) (Tables 2, 3). For C_2+_ hydrocarbons, the isotopic discrepancies between the two methods were consistently within ±0.5‰. These results confirm that the enrichment procedure introduces no measurable isotopic fractionation, supporting its use for PSIA of propane.

**Table 2:** Carbon Isotopic Compositions and Analytical Errors for Laboratory Standards^a^

		δ^13^C (‰)			
Sample	Enrichment method	C_2_H_6_	C_3_H_6_	C_3_H_8_	n
C3	Direct injection			–27.1 ± 0.2	7
	LN_2_/KOH trapping			–26.9 ± 0.3	7
HB	Direct injection	–28.6 ± 0.3	–32.2 ± 0.2	–30.2 ± 0.2	6
	LN_2_/KOH trapping	–28.5 ± 0.3	–31.7 ± 0.3	–29.8 ± 0.2	6

a *n* = number of replicate analyses.

**Table 3:** Position-Specific Carbon Isotope Composition and Analytical Errors for Propane Standards^a^

			Position-specific carbon isotope composition (‰)			
Sample	Enrichment method	δ^13^C_propane_(‰)	δ^13^C_t_	δ^13^C_c_	Δ_C–T_	n
C3	Direct injection	–27.1	–26.7 ± 0.2	–27.8 ± 0.3	–1.1 ± 0.4	3
	LN_2_/KOH trapping		–26.8 ± 0.2	–27.7 ± 0.4	–0.9 ± 0.6	3
HB	Direct injection	–30.2	–30.9 ± 0.1	–28.9 ± 0.2	2.1 ± 0.4	6
	LN_2_/KOH trapping		–31.1 ± 0.2	–28.5 ± 0.3	2.6 ± 0.5	6

a *n* = number of replicate analyses.

This workflow was applied to kerogen-derived pyrolysis gases analyzed in duplicate over four temperature intervals (370–404 °C, 416–466 °C, 476–500 °C, and 516–564 °C), yielding replicate differences of <0.1‰ for δ^13^C_propane_ and δ^13^C_terminal_, and <0.2‰ for δ^13^C_central_ and Δ_C–T_ (Table 4). These deviations were within the precision established with standard gases, indicating that cogenerated pyrolysis products do not introduce detectable isotopic fractionation during enrichment or measurement. At the highest temperature interval, propane yield was lower, and only a single PSIA measurement was obtained. Nevertheless, the consistency of the data set confirms that the workflow maintains reproducibility for low-abundance propane within complex kerogen-derived gas mixtures.

**Table 4:** PSIA of Propane for Kerogen at Different Pyrolysis Temperature and Natural Gas

Sample	Temperature (°C)	δ^13^C_propane_ (‰)	δ^13^C_t_ (‰)	δ^13^C_c_ (‰)	Δ_C–T_ (‰)
YC	370–404	–36.2	–37.1	–34.4	2.7
		–36.1	–37.0	–34.2	2.8
	416–466	–34.2	–36.3	–30.2	6.1
		–34.2	–36.2	–30.3	5.9
	476–500	–28.5	–30.9	–24.2	6.7
		–28.5	–31.0	–24.1	6.9
	514–564	–25.3	–26.3	–23.5	2.8
ZY1 HF	Room temperature	–37.2	–34.5	–42.0	–7.5
		–37.3	–34.6	–42.2	–7.6

A highly mature dry natural gas (ZY1HF; dryness coefficient = 0.99) was selected for testing, with a propane concentration below the detection limit (approximately 2000 ppm). By repeated injections and cryogenic enrichment, propane was enriched from approximately 200 mL of gas for isotope analysis. Duplicate measurements yielded δ^13^C_propane_ of −37.3‰ to −37.2‰, with δ^13^C_terminal_ of −34.6‰ to −34.5‰, δ^13^C_central_ of −42.2‰ to −42.0‰, and Δ_C–T_ of −7.6‰ to −7.5‰ (Table 4). All deviations fell within the established precision of the method. This result indicates that the workflow provides reproducible bulk and position-specific isotope data for natural gas samples with propane concentrations below the detection limit.

### 3.2 Constraining Secondary Cracking of Propane

To constrain the thermal stability of propane under the open-system conditions used in this study, we pyrolyzed pure propane at 300, 500, 520, 550, and 600 °C. Propane remained stable below 500 °C, with no detectable reaction products and a constant residual δ^13^C value of approximately −26.6‰. At 520 °C, low abundances of propylene appeared, accompanied by ^13^C enrichment in the residual propane. At 550 °C and above, wet gas components (ethylene, ethane, and propylene) increased markedly, and δ^13^C_propane_ increased to −25.5‰ (Figure 2, Table S4). These results place the onset of secondary propane cracking at approximately 520 °C under these experimental conditions. The observed isotopic enrichment is consistent with kinetic isotope fractionation during propane cracking.^34^ The experimentally defined threshold provides a reference framework for interpreting the isotopic shifts in the kerogen pyrolysis products. This framework also helps distinguish primary from secondary propane in the following discussion.

**Figure 2:**
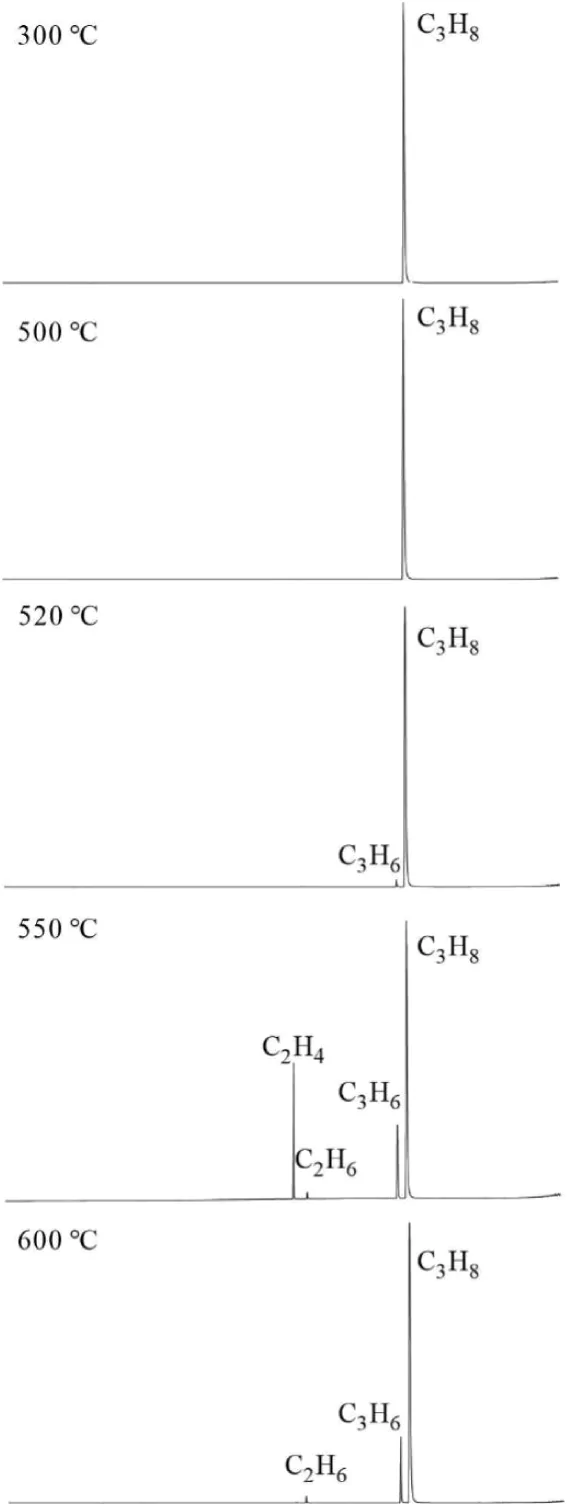
Gas chromatograms of the products from open-system pyrolysis of propane at different temperatures.

### 3.3 Position-Specific Carbon Isotope Systematics of Propane in Open- and Closed-System Pyrolysis

#### 3.3.1 Position-Specific Isotope Signatures

Bulk δ^13^C_propane_ values became systematically enriched with increasing maturity in both open- and closed-system experiments (Table 4, Figure 3a). In contrast, the position-specific parameters exhibited different evolutionary patterns between the two systems (Figure 3b).

**Figure 3:**
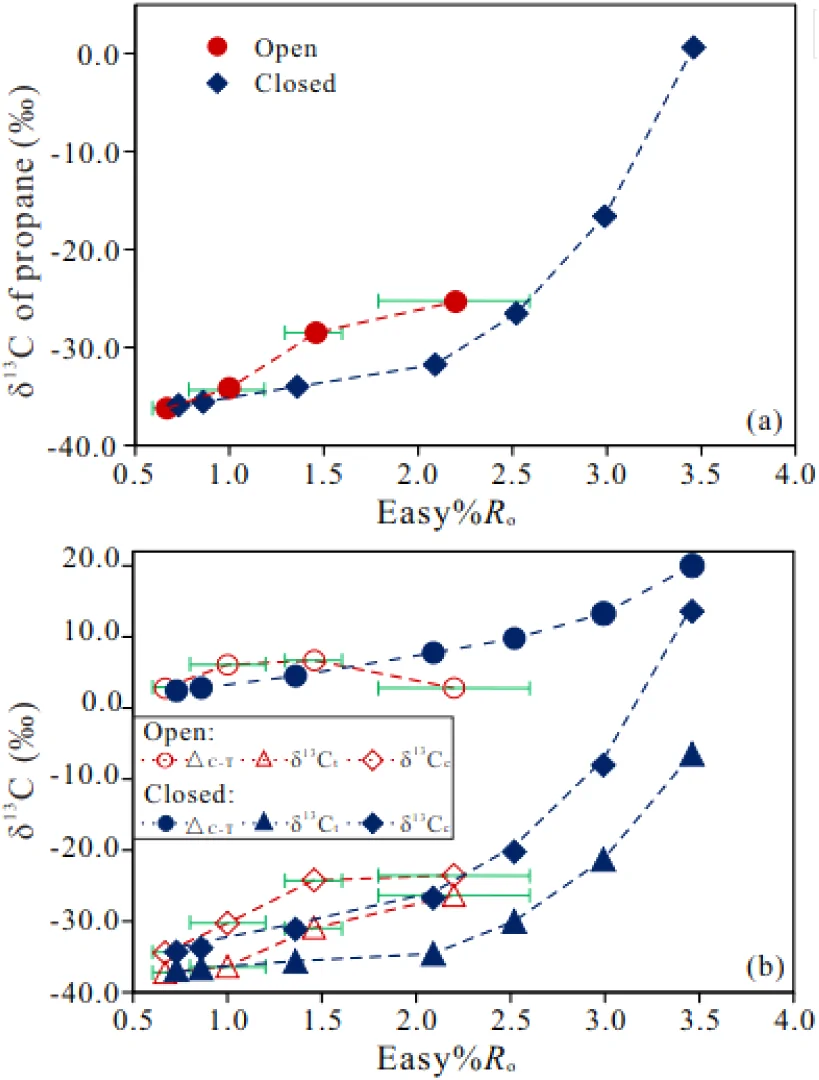
(a) Compound-specific and (b) position-specific carbon isotope compositions of propane versus Easy%*R*_0_ for open- and closed-system pyrolysis of Type II kerogen from the Ordos Basin, China. δ^13^C_t_ and δ^13^C_c_ denote δ^13^C_termina_ and δ^13^C_central_, respectively. For open-system data, each point is plotted at the midpoint Easy%*R*_0_ of four temperature intervals, with horizontal error bars denoting the full maturity range of each interval (see Table S1). Closed-system data represent discrete isothermal pyrolysis points. Dashed lines are visual guides only.

In the open system, the δ^13^C_terminal_ and δ^13^C_central_ values of propane both became progressively enriched in ^13^C with increasing maturity. From 370–404 °C (Easy%*R*_0_ = 0.6–0.7%) to 514–564 °C (Easy%*R*_0_ = 1.8–2.6%), δ^13^C_terminal_ rose from −37.1‰ to −26.3‰, and δ^13^C_central_ rose from −34.4‰ to −23.5‰. Δ_C–T_ exhibited a nonmonotonic trend. It increased from 2.7‰ to 6.1‰ across the 370–466 °C interval, peaked at 6.7‰ within 476–500 °C, and then decreased to 2.8‰ at 514–564 °C. The initial increase in Δ_C–T_ is attributed to preferential cleavage of isopropyl bonds, which is the dominant primary generation pathway at lower maturity. These bonds have lower dissociation energy and yield propane with a ^13^C-enriched central carbon. The subsequent decrease, first observed within the 514–564 °C interval, is consistent with the increasing dominance of n-propyl bond cleavage. This pathway yields propane with a ^13^C-depleted central carbon, thereby lowering Δ_C–T_.^2–4,35^ The 514–564 °C interval encompasses the onset of secondary cracking as constrained by the pure propane experiments (at approximately 520 °C). The Δ_C–T_ decline in the open system therefore records the combined effects of (i) a shift in primary cracking pathways and (ii) incipient secondary cracking of propane.

By comparison, in the closed-system experiments, the δ^13^C_terminal_ values increased continuously from −36.8‰ to −6.4‰, and δ^13^C_central_ values increased from −34.3‰ to 13.6‰ over the entire measured maturity range. The Δ_C–T_ value rose monotonically from 2.4‰ to 20.0‰, with no discernible inflection point. These monotonic trends reflect the cumulative effect of multiple overlapping reactions, including primary cracking of kerogen, secondary cracking of bitumen and crude oil, and subsequent cracking of propane.^5,36^ Therefore, the closed-system PSIA signatures represent a time-integrated bulk signal that inherently obscures the specific contributions of individual primary formation pathways.

The two systems diverge prior to the onset of secondary cracking. The contrasting Δ_C–T_ evolutions suggest that product retention may influence the isotopic signature of propane in the closed system, even before secondary cracking commences, whereas the open-system evolution appears to be governed primarily by the competing bond cleavage pathways described above.

Cross-plot analysis (Figure 4) quantifies the system-dependent differences. In the closed system, the linear relationship between δ^13^C_propane_ and δ^13^C_central_ yielded a slope of 1.30, comparable to the value of 1.31 ± 0.11 reported for hydrous pyrolysis experiments.^36^ For the open system, the corresponding slope was 1.00. A slope of unity suggests that the position-specific isotope composition scales with the bulk composition, consistent with minimal secondary modification. The lower slope observed for the open system therefore indicates that continuous product expulsion attenuates intramolecular isotope redistribution, enabling primary formation signatures to be more clearly resolved. Conversely, in closed-system experiments, product retention results in secondary overprinting that obscures the primary signatures.

**Figure 4:**
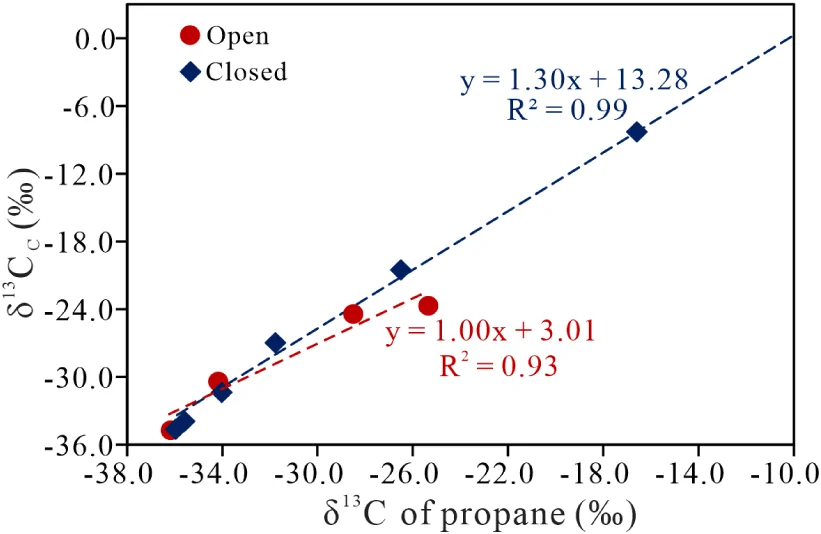
Correlations of δ^13^C_3_ with δ^13^C_c_ of propane from this study.

#### 3.3.2 Geological Implications

The contrasting Δ_C–T_ behaviors suggest that open-system PSIA data more closely approximate primary formation signatures than closed-system data. Accordingly, the open-system trend can serve as a reference framework for distinguishing propane generated predominantly from isopropyl precursors versus n-propyl precursors. The experimentally constrained secondary cracking threshold provides a thermal benchmark for identifying the onset of secondary alteration in natural gas accumulations. Samples with Δ_C–T_ values that deviate from the open-system trend, particularly beyond this threshold, may record secondary cracking or mixing of gases from different sources.

The distinct cross-plot slopes of the open (1.00) and the closed (1.30) systems further suggest that the relationship between bulk and intramolecular isotope compositions can be diagnostic of system openness in sedimentary settings.

## 4 Conclusions

Open-system pyrolysis of a type II kerogen reveals a peak-then-decline evolution in the isotopic difference between the central and terminal carbon atoms of propane (Δ_C–T_). Specifically, Δ_C–T_ increases across the oil window and then decreases at the overmature stage. This trajectory contrasts with the monotonic increase reported for closed systems, suggesting that system openness is a primary control on the intramolecular isotope distribution of propane. Cross-plots of δ^13^C_propane_ versus δ^13^C_central_ yield distinct slopes for open (1.00) and closed (1.30) systems. These distinct slopes support the preservation of primary generation signals under open-system conditions, whereas secondary cracking overprints the intramolecular isotopic signature under closed-system conditions. Pure-propane pyrolysis experiments constrain the onset of substantial secondary cracking at approximately 520 °C (Easy%*R*_0_ = 1.9%), providing a thermal reference for distinguishing primary from secondary overprinting at high- to overmature stages. The offline enrichment protocol enables PSIA of low-abundance propane in natural gas samples with dryness coefficients up to 0.99. These results complement previous closed-system pyrolysis studies and offer a reference framework for interpreting intramolecular isotope signatures of propane. Further validation using natural gas samples from diverse geological settings and maturity ranges is required to refine and extend this framework.

## Supplementary Material



## Notes

Intervals corresponding to Easy%*R*_0_ ranges and midpoint Easy%*R*_0_ values for open-system gas fractions (Table S1); recovery of the LN_2_/KOH trapping system for propane (Table S2); isotopic composition of samples from replicate analyses using direct injection and LN_2_/KOH-trapping methods (Table S3); pyrolysis temperature and corresponding δ^13^C_propane_ (Table S4) (PDF)
