# Supplementary material for: Position-Specific Carbon Isotope Constraints on Propane Formation from Kerogen during Open-System Pyrolysis
Source: ACS Omega. 2026 Oct 12;11(42):65655–61. doi: 10.1021/acsomega.6c08660 (PMC13717661; doi:10.1021/acsomega.6c08660)
Supplement: Supplementary file 1 [file ao6c08660_si_001.pdf]

Position-Specific Carbon Isotope Constraints on Propane Formation from Kerogen during Open-System Pyrolysis

Yuanyuan Ma[1, 2, 3], *, Li Lu [1, 2, 3], Cheng Tao[1, 2, 3], Jipeng Li [1, 2, 3] , Luyun Li[1, 2, 3], Huamin Yang[1, 2, 3] and Yongge Sun[1, 4], *

1 Wuxi Research Institute of Petroleum Geology, Petroleum Exploration and Production Research Institute, SINOPEC, Wuxi, Jiangsu 214126, China

2 State Key Laboratory of Shale Oil and Gas Enrichment Mechanisms and Effective Development, SINOPEC, Beijing, 100083, China

3 Key Laboratory of Petroleum Accumulation Mechanisms, SINOPEC, Wuxi, Jiangsu 214126, China

4 Organic Geochemistry Unit, School of Earth Sciences, Zhejiang University, Hangzhou, Zhejiang 310058, China

* Corresponding authors: mayuanyuan.syky@sinopec.com (Y.M.), ygsun@zju.edu.cn (Y. Sun).

Supporting Information

Table of Contents

Table S1. Intervals corresponding to Easy%R_0 ranges and midpoint Easy%R_0 values for open-system gas fractions

Table S2. Recovery of the LN_2/KOH trapping system for propane (n = 3)

Table S3. Isotopic composition of samples from replicate analyses using direct injection and LN_2/KOH-Trapping methods

Table S4. Pyrolysis temperature and corresponding $\delta^{13}C_{propane}$.

Table S1. Intervals corresponding to Easy%R_0 ranges and midpoint Easy% R_0 values for open-system gas fractions

Temperature interval (°C)	Easy% R_0 (onset)	Easy% R_0 (termination)	Easy% R_0 range	Midpoint Easy% R_0 (used for plotting)
370-404	0.60	0.74	0.60-0.74	0.67
416-466	0.80	1.20	0.80-1.20	1.00
476-500	1.31	1.61	1.31-1.61	1.46
514-564	1.80	2.60	1.80-2.60	2.20

Table S2. Recovery of the LN_2/KOH trapping system for propane (n = 3)

Replicate	Initial volume (mL)	Collected volume (mL)	Recovery (%)
1	1.00	0.85	85
2	1.00	0.90	90
3	1.00	0.90	90
Mean ± SD			88 ± 3
RSD			3.3%

Table S3. Isotopic composition of samples from replicate analyses using direct injection and LN_2/KOH-Trapping methods

Sample	$\delta^{13}C_{ethane}$ (‰)		$\delta^{13}C_{propene}$ (‰)		$\delta^{13}C_{propane}$ (‰)		$\delta^{13}C_t$ (‰)		$\delta^{13}C_c$ (‰)		Δ_{C-T} (‰)	
	DI	LN_2/KOH-T	DI	LN_2/KOH-T	DI	LN_2/KOH-T	DI	LN_2/KOH-T	DI	LN_2/KOH-T	DI	LN_2/KOH-T
C3					-27.0	-27.1	-26.9	-26.6	-27.5	-28.1	-0.6	-1.5
					-27.4	-27.1	-26.7	-26.9	-27.8	-27.5	-1.1	-0.6
					-27.0	-27.1	-26.6	-27.0	-28.1	-27.4	-1.5	-0.4
					-27.0	-26.7						
					-26.9	-27.1						
					-27.0	-26.5						
					-27.2	-26.7						
HB	-28.7	-28.9	-32.2	-31.9	-30.2	-29.5	-31.0	-30.9	-28.7	-29.0	2.4	1.9
	-28.9	-28.8	-32.0	-31.9	-30.5	-29.8	-31.1	-31.1	-28.6	-28.5	2.5	2.6
	-28.2	-28.3	-32.2	-31.4	-30.1	-29.7	-30.8	-31.1	-29.2	-28.5	1.6	2.6
	-28.8	-28.3	-32.4	-31.9	-30.2	-29.9	-31.0	-31.3	-28.8	-28.2	2.2	3.1
	-28.4	-28.4	-32.4	-31.4	-30.3	-29.7	-30.8	-31.4	-29.1	-28.1	1.8	3.3
	-28.8	-28.3	-32.1	-31.6	-30.1	-30.0	-30.9	-31.0	-28.9	-28.7	2.0	2.4

* DI = Direct Injection; LN_2/KOH-T = LN_2/KOH Trapping

Table S4. Pyrolysis temperature and corresponding $\delta^{13}C_{propane}$

Temperature (°C)	$\delta^{13}C_{propane}$ (‰)	
300	-26.6	-26.8
500	-26.6	-26.3
520	-26.3	-26.2
550	-25.5	-25.4
600	-25.4	-25.5
